# Introduction to the AQ-WATCH Project and the AQ-WATCH Toolkit to fight air pollution

**DOI:** 10.1093/eurpub/ckac131.156

**Published:** 2022-10-25

**Authors:** C Li, G Brasseur, C Granier, M Sofiev, R Timmermans, S Basart, G Pfister, R Kumar, B Caillard, Y Boose

**Affiliations:** 1 Environmental Modeling, Max Planck Institute for Meteorology, Hamburg, Germany; 2 Atmospheric Chemistry Observations and Modeling, National Center for Atmospheric Research, Boulder, USA; 3 Laboratoire d'Aérologie, CNRS-Université de Toulouse, Toulouse, France; 4 NOAA Chemical Sciences Laboratory, CIRES, University of Colorado, Boulder, USA; 5 Atmospheric Composition Modelling Group, Finnish Meteorological Institute, Helsinki, Finland; 6 Climate, Air and Sustainability Department, The Netherlands Organisation for Applied Scientific Research, Utrecht, Netherlands; 7 EaAtmospheric Composition Group, Barcelona Supercomputing Centre, Barcelona, Spain; 8 INERIS DEVELOPPEMENT SAS, Verneuil-en-Halatte, France; 9 BreezoMeter Ltd, Haifa, Israel

## Abstract

**Background:**

WHO states that 9 out of 10 persons in the world do not breath clean air and 8 million people die prematurely from air pollution each year. The problem is well understood, but actions to mitigate it are lacking. The purpose of the EU-funded AQ-WATCH Project is precisely to develop effective tools based on the most advanced science technologies to help decision-makers in government and the private sector to address air pollution issues in regions of the world where they operate.

**Objectives:**

AQ-WATCH aims to develops a supply chain to generate innovative downstream products for improving air quality forecasts and attribution based on existing space/in-situ observations to improve public health and to optimize renewable energy in regions of the world. The project consortium includes research and business-oriented partners, who brings together the required expertise to define the optimal functionalities of these products to bring them to the market.

**Results:**

The AQ-WATCH products are organized into 5 modules: (1) Air quality atlas, (2) Air quality attribution & mitigation, (3) Dust and fire forecast, (4) Fracking analysis, and (5) Air quality forecast. They are developed for 3 target regions (Beijing, Colorado and Santiago de Chile) and are integrated into a unified user-interface, the AQ-WATCH Toolkit. Product developers and prime users in the target regions are constantly interacting, and the user feedback is collected, analyzed and included during the product development.

**Conclusions:**

Collaborative work done in AQ-WATCH shows strategic interaction between our research and business-oriented partners. Contributions from local parties are proven to be valuable for regional adaption of the products. A throughout dissemination including regional workshops is essential to ensure proper knowledge uptake by the target audience. Constant exchange with the private sector is required for a smooth transfer from scientific results to commercialized marketable products.

**Key messages:**

• The AQ-WATCH Project follows EU’s initiative to utilize its space observations with added values to develop easily-accessible tools to fight air pollution applicable to regions of the world.

• The AQ-WATCH Toolkit is developed with iterative feedback exchanges between product developers and local users to address air pollution issues, and will be eventually exploited to the market.

